# Efficacy of a Social Self-Value Empowerment Intervention to Improve Quality of Life of HIV Infected People Receiving Antiretroviral Treatment in Nepal: A Randomized Controlled Trial

**DOI:** 10.1007/s10461-016-1546-z

**Published:** 2016-09-09

**Authors:** Dharma Nand Bhatta, Tippawan Liabsuetrakul

**Affiliations:** 1 Department of Public Health, Nobel College, Pokhara University, Kathmandu, Nepal; 2 Faculty of Medicine, Epidemiology Unit, Prince of Songkla University, Hat Yai, Thailand

**Keywords:** HIV, Stigma, Quality of life, Social support, Empowerment, ART, Intervention, Adherence

## Abstract

We developed a comprehensive and culturally applicable empowerment intervention social self-value package with an aim to assess its efficacy in order to improve the quality of life (QoL) of HIV infected people receiving antiretroviral treatment. Participants were randomly allocated to receive either six weekly intervention sessions or standard care. Nonlinear mixed-effects models were performed to compare changes in empowerment scores over time. Between September and November 2014, 1447 individuals were screened, of whom 132 were randomly assigned to either the intervention or control group. The mean scores of empowerment, social support and quality of life increased and stigma scores were reduced in the intervention group at 3- and 6-months. An intervention effect on social support, stigma and QoL was significantly increased by time and group with low and high empowerment. No adverse events were reported. The empowerment intervention was efficacious in improving QoL of HIV infected people.

**Electronic supplementary material:**

The online version of this article (doi:10.1007/s10461-016-1546-z) contains supplementary material, which is available to authorized users.

## Resumen

Hemos desarrollado un fortalecimiento completo y cultural applicable a la intervenci**ó**n social del paquete del valor propio con la intención de evaluar su eficacia para mejorar la calidad de vida de las personas infectadas por el VIH que están recibiendo ART. A los participantes se les adjudicó aleatoriamente la asignación de seis dosis semanales o los cuidados estandar. El resultado de los efectos se presentó para comparar los cambios en los valores del fortalecimiento a lo largo del tiempo. Entre septiembre y noviembre de 2014, 1447 individuos fueron moritonizados, de los cuales 132 fueron aleatoriamente asignados para cada intervención o grupo de control. La media del valor del fortalecimiento, apoyo social y calidad de vida incrementaron y los valores del estigma fueron reducidos en la intervención grupal entre 3 y 6 meses. Los efectos de una intervención al apoyo social, estigma y calidad de vida se incrementaron significativamente en ese periodo y el grupo con un bajo y alto fortalecimiento. No hubo efectos secundarios notificados. La intervención en el fortalecimiento fue satisfactoria en la mejora de la calidad de vida de la gente infectada por el VIH.

## Background

Epidemiological studies have highlighted decreases in HIV incidence and increased deaths related to HIV/AIDS [1–5]. However, the prevalence of HIV has remained extensive and epidemic contained enormous heterogeneity [4]. Decreasing trends of epidemics and increased life expectancy of people infected with HIV have been reported but risky sexual behaviors have promoted the transmission of HIV among general populations, and re-infections and co-infections among HIV infected people [3, 6]. Disease burdens are reported to be more common among HIV infected populations compared to general populations, altering their quality of life (QoL) [7–9]. The life expectancy among people living with HIV after they have initiated suitable antiretroviral treatment (ART) or combined antiretroviral treatment is reported to be similar to the general population [10, 11].

Availability of the ART among HIV infected people is very low and combined with psychological distress might lead to the development of anxiety, low QoL and increased stigma [4, 12]. The effect of ART on QoL was found to be reasonable among HIV infected people [13–15]. Further, their quality of life is influenced by health status, economic factors and psychological status [16]. The mechanism of how immunological and virological response influences quality of life among HIV infected people has not been comprehensively studied and therefore the effect of HIV status on QoL is unclear. Albeit, much less has been identified about the QoL of HIV infected people in comparison with other people despite considerable progress in medical prospective [16, 17]. Moreover, the effect of treatment on HIV might be affected by several factors which would help to increase stigma, reduce QoL and social support and disempowerment of HIV infected people [18–21].

Social taboos and stigma are universal socio-cultural barriers for HIV control and prevention [22, 23]. Empowerment and social support could be helpful assets among HIV infected people to enhance their QoL, reduce stigma and improve adherence to ART in resource poor settings [24, 25]. A practical and integrated program is needed for empowerment of HIV infected people [26, 27]. Empowerment would be best approached to reduce HIV risk associated problems with cost effective interventions [28–30]. However, HIV related empowerment based programs are sparse and it has been necessary to strengthen and implement them with usual ART and other programs with political and social transforms [26, 31–33].

Different organizations have set the goal to achieve zero discrimination, transmission and stigma but the interventions are too sparse to cover these goals [2, 34, 35]. In addition, the ‘90-90-90’ target has been set by UNAIDS by the year 2020 [36]. Therefore, extensive socially and culturally accepted cost-effective interventions are needed which enhance QoL and social support, and eliminate stigma among HIV infected people in resource poor settings [15, 37–40]. Unfortunately, most of the interventions were established in developed countries and few from developing countries. There is thus an urgent need to develop a culturally sensitive intervention for use in developing countries. An empowerment intervention program we have developed, designed to improve QoL of HIV infected people, was developed on the foundation of the diffusion model of innovations [41], and followed different theories to connect empowerment framework that assumed to change behavior, self-esteem, social support, discrimination, stigma and QoL [6, 42–52]. The aim of this study was to assess the efficacy of a social self-value empowerment intervention package to improve QoL of HIV infected people receiving ART. Furthermore, we assessed the effect of this intervention to enhance social support and reduce stigma.

## Methods

### Study Design, Settings and Participants

**Fig. 1:**
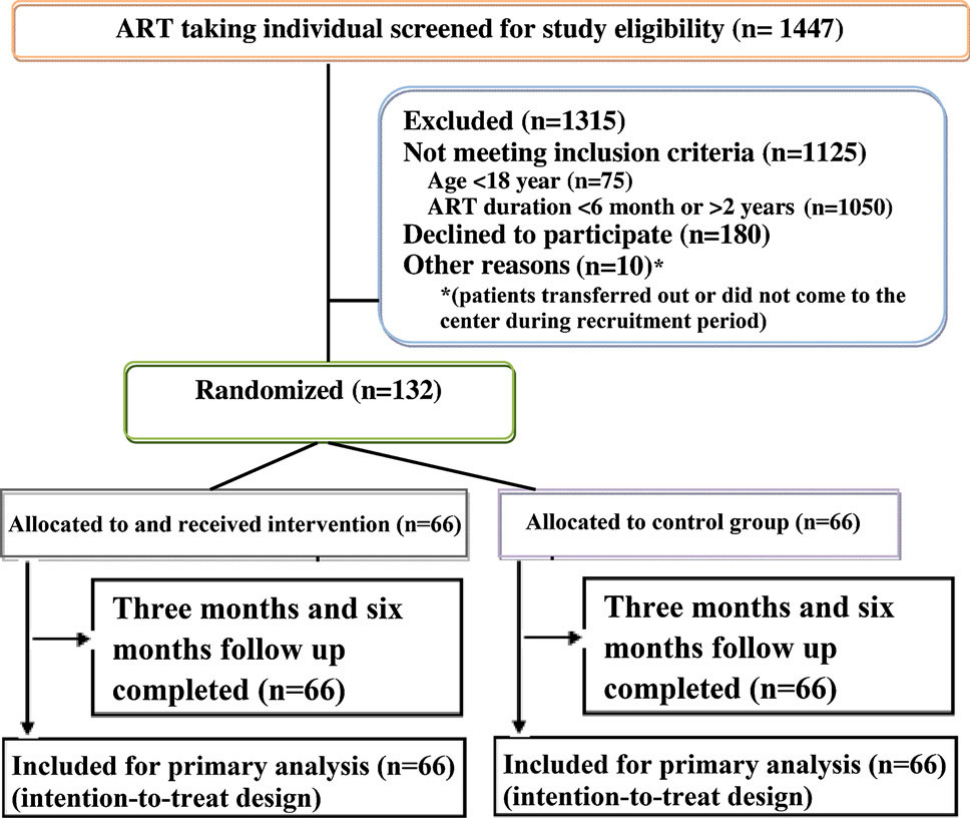
Study design and participant enrolment flow diagram

In this open label, parallel, randomized controlled trial, HIV infected participants receiving ART from the ART center in Kathmandu, Nepal were recruited. The study was carried out between September 2014 and June 2015 in Sukraraj Tropical and Infectious Disease Hospital (STIDH), Teku, Kathmandu, which is administered by the National Center for AIDS and STD Control (NCASC) [53]. STIDH is the largest ART center in the country that has been providing multidisciplinary medical services for all HIV infected people since 2004 [54]. Details of the study participants recruitment and design are presented in Fig. 1.

To be included in the study, participants had to be HIV infected, aged 18 years or older, and have been receiving ART between 6 months and 2 years prior to the study as per the national ART guidelines of NCASC [55]. Participants with severe health problems (including psychotic disorders, visual and hearing problems), had attended similar intervention programs or any other education programs, were unable to attend all the study follow up visits, or were unwilling to disclose their HIV status among other participants were excluded from the study.

We calculated that 132 participants (66 in each group) would achieve 80 % power to detect 20 % mean difference in QoL scores between the two groups with a confidence interval of 95 %.

### Randomization and Masking

Eligible participants were randomly allocated (1:1) to receive either the intervention sessions or standard care. Randomization was performed by a random number generator with permuted blocks of six. Allocation concealment was done by using sequentially numbered opaque sealed envelopes. The random number sequence was generated by an independent data manager. Members of the research team and participants were masked to these numbers and the randomization process. None of the participants were allowed to modify their assignments after randomization. The statistician and research staff doing the baseline and follow up assessments were masked to assignment of participants by using a unique code system. Enrollment, randomization and intervention sessions were conducted between September and November 2014. First follow up assessments were done after 3 months from baseline (January–February, 2015) and 6 months follow-up assessments were done 3 months from the first follow up (May–June, 2015).

### Intervention Procedures

Baseline information was collected after recruitment and allocation of the participants. The intervention was delivered over six sessions held weekly at the ART center lasting one and half hours. Sessions were conducted with a group of 8–10 participants. All the intervention sessions were facilitated by two national level trainers with a public health graduate degree. A facilitator delivered the intervention with participatory learning activities, buzz sessions, brain storming, lecture, and discussion techniques. Participants were encouraged and motivated to communicate and discuss with different people about prevention, treatment and disclosure of HIV issues [56, 57].

The development of the intervention contents involved review of existing literature that followed social learning and action theory and empowerment principles for HIV prevention and treatment [6, 42–52]. Culturally accepted and adopted components were developed after several consultations with experts and pre-tested among HIV infected people. Based on the findings from consultants and pre-testing, a complete manual for execution of a 6 week group intervention was developed by the research team. The empowerment intervention mainly focused on autonomy and community activism, self-esteem/self-efficacy, self-care, optimism and control over the future, family and social relationships, power-powerlessness, management of stress and righteous anger, stigma and discrimination issues, legal provisions, and human and health rights. Details of the empowerment intervention contents are available in Additional Table 1.

**Table 1:** Clinical and behavioral characteristics

	Control group (*n* = 66)	Intervention group (*n* = 66)	*p* value
Age at HIV diagnosis			
Median (IQR)	33 (26.5, 41)	33 (30, 40)	0.56
≤33 years	34 (51.5)	34 (51.5)	0.10
>33 years	32 (48.5)	32 (48.8)	
Age at ART initiation			
Median (IQR)	35 (28, 42.8)	35 (30.2, 40.8)	0.55
≤35 years	36 (54.5)	38 (57.6)	0.86
>35 years	30 (45.5)	28 (42.4)	
Duration of ART			0.60
<1 year	38 (57.6)	34 (51.5)	
≥1 years	28 (42.4)	32 (48.5)	
Spouse HIV status			0.15
Negative	16 (27.6)	26 (41.9)	
Positive	42 (72.4)	36 (58.1)	
Mode of HIV transmission			0.16
Others	34 (51.5)	25 (37.9)	
Sex-worker	32 (48.5)	41 (62.1)	
Sexual intercourse in last 3 months			1.00
Yes	50 (75.8)	50 (75.8)	
No	16 (24.2)	16 (24.2)	
Extra-marital sex			0.78
Yes	7 (14)	9 (18)	
No	43 (86)	41 (82)	
Clinical stage			1.00
I and II	34 (51.5)	35 (53)	
III and IV	32 (48.5)	31 (47)	
Known co-morbidities			0.80
Tuberculosis	10 (15.2)	8 (12.1)	
Other	56 (84.8)	58 (87.9)	

Briefly, all intervention sessions were started with group and ground rules, formal opening and closing custom, sharing and discussion. The first session started with rapport building, emotions, sharing uncomfortable situations and management of negative feelings and anger. The second session focused on barriers and strategies of HIV disclosure, self-esteem/self-respect/self-worth, stigma and defeat with stigma. The third session involved discussions about healthy body with healthy mind, healthy sexual relations, means to be HIV infected or non-infected and to be a man or woman, optimism and control over the future, sexuality, adherence of ART and other treatment and prevention options. The fourth session involved educated strategies for planning healthy relations with family members, the community and society, ways of effective communication and maintaining healthy relations, autonomy and community activism, and roles and responsibilities in the society. The fifth session involved education about the effects of alcohol consumption, drug use, smoking, developing skill to prevent co-infection, re-infection and risky sexual behavior, diet and exercise. In the sixth session, participants were educated about legal empowerment, human rights, legal protection, powerlessness, discrimination, stress, freedom of voice against discrimination, health rights and future goals [56].

Fidelity of the intervention was maintained with continuous monitoring of the allocated time for topic, methods and contents of the sessions by a health officer and supervised by the research team leader. Participants were assured to receive equal chances on discussion with privacy. A checklist was developed to maintain fidelity of the intervention. The checklist included intervention contents (each session had different contents), time allocated for each activity, participants interaction with listening, openness, attentiveness, engagement, understanding and reinforcement and an agenda for the next session. The percent of items rated as “appropriate” by the reviewer was more than 95 %, however we had only one reviewer due to limited manpower in the government agency so we could not calculate the level of agreement. A debriefing session was conducted at the end of each session for the feedback from the reviewer and facilitators. Intervention sessions were not gender-separated. We measured the acceptability using a session evaluation form (SEF) [58] and satisfaction of the participants in the intervention using a client satisfaction questionnaire (CSQ-8) [59] at the end of the intervention. All the items in the SEF indicated a higher level acceptability among the intervention group. The mean score of all items ranged from 3.68 to 3.82 with a standard deviation of 0.39 to 0.47. The total score indicated that participants either agreed or strongly agreed with the sessions. The level of participant satisfaction was high. All the participants stated that the quality of the intervention was excellent. The majority of participants (92.4 %) were very satisfied with the amount of help provided to them. Almost all (95.5 %) agreed to join the program again and were willing to refer it to others (data not shown).To maintain compliance, at the end of each session the counselors motivated participants to participate in the next session, encouraged voluntary independent participation and provided gift vouchers. The overall retention rate was 96.6 % in the intervention session. All the participants were compensated for each of their six sessions with an equivalent of USD 20.

### Standard Care

All participants received routine standard care as per the NCASC guidelines [55]. This included pre ART counseling, routine medical and laboratory tests and monthly follow up for ART. Standard care in Nepal is provided by government organizations and ART is dispensed free of charge.

### Study Procedures

Participants were asked to provide information on clinical and behavioral characteristics, QoL, stigma, social support and empowerment at baseline, and at the scheduled 3- and 6-months follow up visits. To minimize data entry errors and enhance quality control, double data entry was employed and extensively supervised by the research team leader. Anonymity and confidentiality were maintained with assigned unique codes at randomization, baseline and follow up. The intervention protocol and tools were pre-tested among ten HIV infected people before data collection. All tools were translated into Nepali and back translated into English and appeared culturally suitable to the experts.

### Outcomes

Background, behavioral and clinical characteristics of the participants were collected including age at HIV diagnosis, age at ART initiation, HIV status of spouse, mode of HIV transmission, sexual intercourse in the last 3 months and extra-marital, clinical stage, co-morbidity, adherence to ART (coded as yes or no) and empowerment. The primary outcome was QoL. Secondary outcomes were stigma and social support.

QoL was measured using WHOQoL-HIV [60] which contains 29 items divided into six domains, namely physical, psychological, level of independence, social, environmental and spiritual. It has also one general item score that measures overall quality of life and general health. All the items were rated using a 5-point Likert scale where 1 indicated low or negative perceptions and 5 indicated high or positive perceptions. Higher scores indicated better quality of life. All the domain scores were obtained by adding the component means in the individual domain, and dividing by the number of components in that domain, and multiplying by 4, so that scores ranged from 4 (worst possible QoL) to 20 (best possible QoL).

Social support was measured using the social support questionnaire number (SSQN) and social support questionnaire satisfaction (SSQS) scales [61]. SSQN indicates number of supportive persons and SSQN indicates satisfaction with available social support. Both domains included six questions. The SSQN collected the number of supportive persons that denotes different types of social support. The SSQS were rated using a 6-point Likert scale ranging from very dissatisfied to very satisfied with available support. Higher SSQN scores indicated a perceived higher level of supportive persons and higher SSQS scores indicated higher level of satisfaction from available support.

Stigma was measured using a 23-item scale questionnaire [62]. Each item was rated using a 4-point agreement scale ranging from strongly disagree to strongly agree. Total stigma scores ranged from 23 to 92. There were three subscales, namely shame/blame/social isolation (10–40 score), perceived discrimination (8–32 score) and equity (5–20 score).

Empowerment was measured using a 28-item scale questionnaire [63] containing a 4-point agreement scale ranging from strongly disagree to strongly agree. Total scores ranged from 28 to 112. Empowerment was then classified into low or high using the first quartile score as the cut-off value to ensure the sample sizes were adequate.

### Statistical Analyses

Each study group’s clinical and behavioral characteristics measured were initially compared using Chi square tests or Fisher’s exact test for categorical outcomes and Wilcoxon’s signed rank test and unpaired *t*-tests for continuous outcomes, as appropriate.

Nonlinear mixed-effects regression models were used to evaluate the effect of the intervention on the primary and secondary outcomes. Covariates included empowerment, adherence to ART, age, sex, time (baseline, 3-, or 6-months follow-up), and group-by-time interactions. The mixed effect model was used to adjust for the underestimation of variances in analysis for longitudinal data [64]. The effects of empowerment, social support and stigma on the QoL were also analyzed with a mixed effect model.

Nonparametric mixed-effects regression models were also used to evaluate the relative intervention effects on social support, stigma and QoL with and without stratification by empowerment. Relative intervention effects with 95 % confidence intervals with and without stratification by empowerment level among both intervention and control groups at baseline, 3- and 6-months follow up were presented. Estimated improvements with 95 % confidence intervals were plotted.

All statistical analyses were conducted with R software [65]. *p* values less than 0.05 were considered to be significant.

### Ethical Considerations

Extensive anonymity, confidentiality and privacy were maintained during the recruitment, intervention and data collection process. Confidentiality and safety of the intervention study data were maintained as per the standard protocol [66]. National guidelines and principles of Nepal health research council, and the declaration of Helsinki were followed to obtain written informed consent and enrollment of the participants. Participants were fully informed about time, methods, and their right to withdraw at any time and skip any question for any reason. Reimbursement for travel cost during the intervention and follow up period were provided to all the participants. No conflict of interest and no direct or indirect financial benefits were anticipated by researchers in this intervention.

Research Ethics Committee, Faculty of Medicine, Prince of Songkla University, Thailand (57-0146-18-5) and Institutional Ethical Review Committee of STIDH, Nepal (063/071/72) approved this study. The trial was registered through Thai Clinical Trial Registry with registration number TCTR20140814002 (www.clinicaltrials.in.th).

## Results

A total of 1447 HIV infected people receiving ART were screened from September to November 2014, of which 1135 were ineligible due to age <18 years (*n* = 75), duration of ART <6 months or >24 months (*n* = 1050), and others (*n* = 10). 180 eligible participants refused to join the study, giving a response rate of 42.3 %. Finally, 132 participants were recruited and randomly assigned to the control group (*n* = 66) or intervention group (*n* = 66).The diagram of participant flow is presented in Fig. 1. All the recruited participants completed the study at baseline, 3- and 6-months follow up. The overall retention rate was 96.6 %. No unfavorable events were reported during the study period.

The mean age of the participants was 36.1 (SD = 7.8) years. Most of the participants were female (53 %), non-indigenous (56.1 %), could read and write only (28.8 %), and were married (74.2 %). The majority (84.8 %) of participants had an average of two children and there were no significant differences at baseline between the control and intervention groups. Table 1 compares baseline behavioral and clinical characteristics between participants in the two groups. More than 50 % of participants were diagnosed with HIV before 33 years of age among both control and intervention groups. Two thirds of participants (62.1 %) in the intervention group were infected through sexual contact. Three fourths (75.8 %) were sexually active within the last 3 months. No significant differences of behavioral and clinical characteristics were detected between the two groups.

**Fig. 2:**
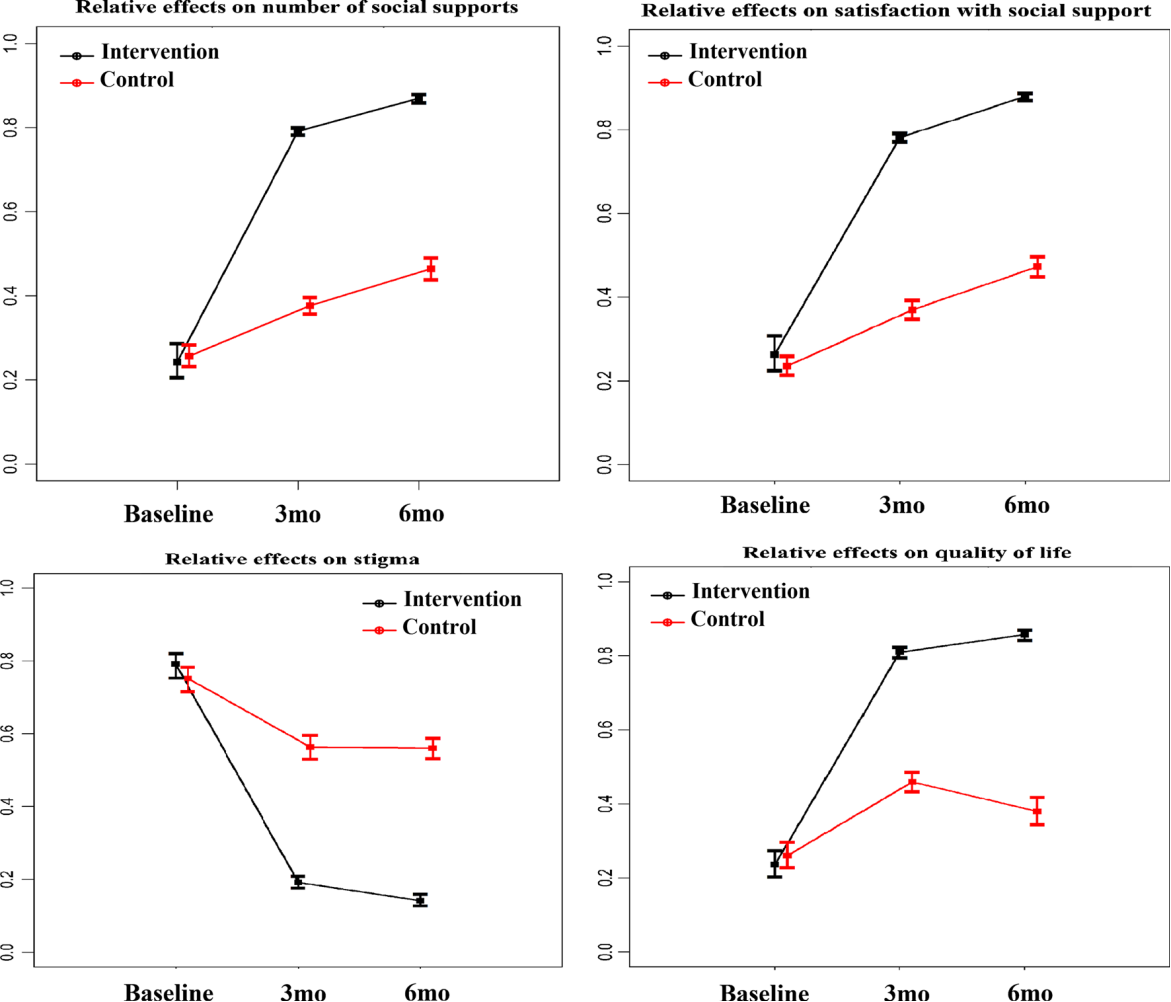
Relative intervention effects on social support, stigma and quality of life using nonlinear mixed-effect model. All *p* values for time trend between intervention and control on each outcome were significantly different with *p* < 0.001, *3mo* three months follow up, *6mo* six months follow

**Fig. 3:**
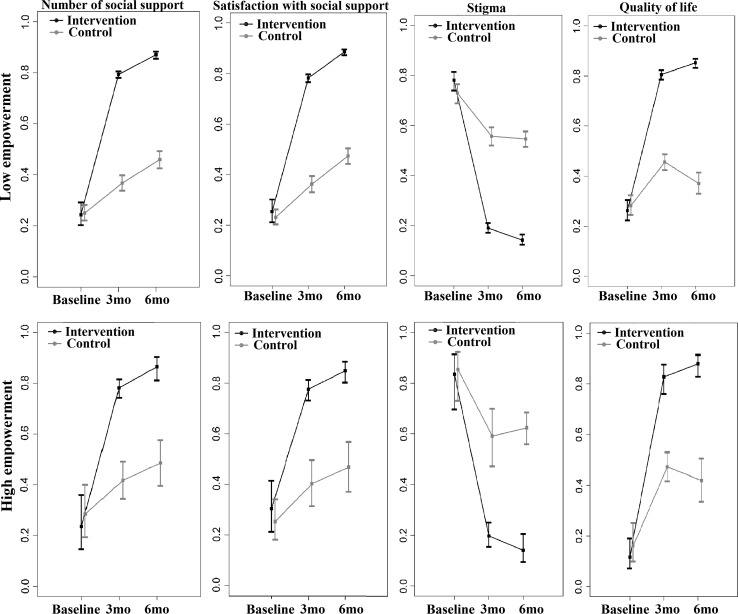
Relative intervention effects on social support, stigma and QoL stratified by empowerment using nonlinear mixed-effect model. All *p* values for time trend between intervention and control on each outcome were significantly different with *p* < 0.001, *3mo* three months follow up, *6mo* six months follow up

The mean empowerment, social support, stigma and QoL scores are presented in Table 2. All the scores at baseline were equally static in both groups. The mean scores of empowerment and QoL increased two-fold in the intervention group at 3 months but no further increase at 6 months. Stigma scores were reduced by half at 3 months in the intervention group with no further changes at 6 months. Social support scores increased by 1.5 times higher at 3 months follow up in the intervention group compared to the control group. Overall mean QoL scores at 3 months increased by 80 % in the intervention group. The relative intervention effects among total social support with number (TSSQN), total social satisfaction with support (TSSQS), stigma and QoL had a similar trend in the intervention group (Fig. 2). Minimal improvements of outcomes were observed in the control group. Figure 3 reveals the relative intervention effects of social support, stigma and QoL by level of empowerment. The effects of the intervention on social support, stigma and QoL persisted at 6 months regardless of level of empowerment (high vs. low).

**Table 2:** Mean values of empowerment, stigma, social support and quality of life scores among HIV infected people

	Baseline		3 months follow up		6 months follow up	
	Control	Intervention	Control	Intervention	Control	Intervention
Empowerment	46.70	46.38	48.23	94.68	46.53	95.92
Social support						
TSSQN	15.04	14.70	16.92	28.09	18.58	30.70
TSSQS	15.04	15.42	17.44	29.00	19.44	32.67
Overall stigma	76.03	76.50	72.91	39.41	73.03	38.26
Shame/blame/social isolation	33.11	33.64	32.21	16.89	31.27	16.76
Perceived discrimination	26.00	26.17	24.36	14.45	24.95	13.86
Equity	16.92	16.70	16.33	8.06	16.80	7.64
Overall quality of life	7.76	7.68	8.46	15.47	8.15	15.81
Physical	8.06	7.89	8.59	15.74	8.23	15.88
Psychological	7.78	7.76	8.10	16.04	8.05	15.99
Independence	7.42	7.32	8.58	15.51	8.32	15.65
Social relations	7.61	7.53	8.74	15.47	8.17	15.82
Environment	7.48	7.48	8.20	15.14	7.98	15.68
Spiritual/religious/personal belief	8.45	8.27	8.88	15.12	8.39	15.92
General overall health	11.97	11.58	10.64	13.76	9.88	15.03

*TSSQN* total social support with number, *TSSQS* total satisfaction with social support

Table 3 presents the outcomes from the nonlinear mixed-effects regression model. Empowerment significantly reduced stigma and increased QoL (*p* < 0.001 and *p* < 0.001) after adjusting for age, gender, adherence to ART, group and time. There was no significant difference in any outcome between the intervention and control group at baseline. There were significant interaction effects of intervention by time indicating that improvements in social support and QoL for the intervention group were significantly higher compared to the control group over time (*p* < 0.001), while stigma was significantly lower (*p* < 0.001). Estimated differences in improvement in social support, stigma and QoL at 3- and 6-months from baseline between intervention and control were significant (all *p* values < 0.001). Increasing ART adherence was associated with a reduction of stigma (*p* < 0.001). Age and gender were not significantly associated with any outcome.

**Table 3:** Effect of empowerment intervention on social support, stigma and quality of life using nonlinear mixed-effects regression model

Parameter	TSSQN			TSSQS			Stigma			Quality of life		
	Estimate	SE	*p* value	Estimate	SE	*p* value	Estimate	SE	*p* value	Estimate	SE	*p* value
Empowerment	−0.010	0.038	0.795	−0.024	0.040	0.544	−0.127	0.038	<0.001	0.057	0.009	<0.001
Age	−0.001	0.028	0.997	−0.038	0.029	0.196	0.005	0.016	0.744	0.005	0.005	0.289
Male versus female	1.196	0.449	0.008	1.215	0.464	0.009	0.315	0.250	0.208	−0.009	0.074	0.896
ART adherence	−0.681	0.393	0.083	−0.424	0.409	0.300	−1.300	0.402	<0.001	0.145	0.105	0.151
Intervention versus control at baseline	−0.496	0.532	0.351	0.243	0.550	0.659	0.388	0.425	0.360	−0.060	0.109	0.579
Time (months)			<0.001			<0.001			<0.001			<0.001
Intervention × Time			<0.001			<0.001			<0.001			<0.001
Difference in improvement from baseline between intervention and control group												
3 months follow up	11.983	1.868	<0.001	12.304	1.936	<0.001	−28.028	1.890	<0.001	4.430	0.474	<0.001
6 months follow up	12.967	1.977	<0.001	14.041	2.049	<0.001	−28.927	1.998	<0.001	4.904	0.501	<0.001

*SE* standard error, *TSSQN* total social support with number, *TSSQS* total satisfaction with social support

Predictors of overall QoL are presented in Table 4. Increased empowerment had a significantly higher level of QoL (*p* < 0.001). Increased stigma had a lower level of QoL but this was not statistically significant. Social support had no significant effect on QoL. Improvement in QoL remained statistically significant at 3- and 6-months follow up (*p* < 0.001).

**Table 4:** Prediction of QoL by intervention group after adjusting for level of empowerment, social support and stigma using nonlinear mixed-effects regression model

Parameter	Quality of life		
	Estimate	SE	*p* value
Empowerment	0.057	0.009	<0.001
TSSQN	0.007	0.012	0.574
TSSQS	−0.009	0.011	0.410
Stigma	−0.004	0.012	0.771
Intervention effect at baseline (intervention vs. control)	−0.051	0.108	0.371
Time (in months)			<0.001
Intervention × time			<0.001
Estimated difference in improvement in QoL from baseline (intervention vs. control)			
3 months follow up	4.310	0.610	<0.001
6 months follow up	4.788	0.642	<0.001

*SE* standard error

## Discussion

Empowerment, social support, stigma and QoL of HIV infected people at baseline were low among both groups. The improvement of social support, stigma and QoL was seen immediately after 3 months among those in the intervention group and persisted for another 3 months. The intervention positively affected social support, stigma and QoL in the equivalence regardless of level of baseline empowerment. Empowerment significantly affected social support, stigma and QoL but only empowerment was shown to be a significant predictor of QoL in addition to the intervention.

Clinical and behavioral characteristics were not significantly different at baseline between the two groups. Recruitment process followed strong protocol that assured the high retention rate in the intervention and lower loss to follow up. Most eligible participants declined to participate in the study; however, there was no difference in their background characteristics compared to those who participated in the study. Reasons for refusal to participate in the study were: too busy, unable to manage time for all the intervention sessions, lack of interest in the study and unavailability for follow up.

Social support was significantly improved by enhancing empowerment in the intervention group. As far as in our knowledge, empowerment interventions for all the HIV infected population were not available and comparison of the results with this trial would be difficult. A randomized controlled trial using a group support psychotherapy for HIV infected people as an intervention and measured at the same period as our study also showed an increasing social support after intervention by time [67]. Slightly improved social support in the control group at 3 months might be explained by learning process of participants from the repetitive questionnaires inducing their behavior changes (pretest sensitization effect) and possible effects of the contamination [68]. Stigmatization, discrimination, and cognitive state—the psychological condition that is characterized by a lack of obvious and logical belief and behavior—might be removed with increased social support through empowerment.

Overall stigma decreased after the intervention, more so in the intervention group. This finding was supported by the conclusion of systematic reviews that focused on any interventions for stigma reduction [69, 70]. Further, it has been suggested that the limited interventions were available to combat different forms of stigma and discrimination experienced by HIV infected people [70]. Therefore, our study applied an empowerment strategy to overcome and resist the manifestation of discrimination and stigma among HIV infected people with the adaptation of local context and culture. In addition, empowerment would help to defeat symptoms of stigma among HIV infected people and our trial found that increased empowerment could significantly reduce stigma. This result was similar to a systematic review and meta-synthesis which highlighted increased adherence were linked with decreased stigma [71].

The intervention was found to be significantly effective for improving QoL. Systematic reviews based on different interventions and observational studies which focused on QoL of HIV infected populations showed inconclusive results [72–75]. This might be due to the use of different measurement scales, sampling process and sample size, culture and context of study settings in different studies. Therefore, we used different analytical approaches and pretested cultural and contextual appropriateness of the intervention manual which could improve the reliability of the outcome.

Existing cross-sectional studies from China suggested that social support and stigma were correlated with QoL and social support was the moderator of the impact of stigma on QoL of HIV infected people [76, 77]. A systematic review revealed that social support and ART adherence were associated with QoL of HIV infected people [78]. However, in this study, social support, stigma and ART adherence were not statistically significant but increased stigma showed negative effects on QoL. The small sample size could be the possible reason that we could not established statistically significant results with these variables.

This study has several strengths. First, this randomized controlled trial was based on real world study settings that represent the ART receiving HIV infected individuals. Second, the intervention attendance and retention rate was high which signifies the feasibility and acceptability of the intervention for HIV infected people. Third, this is the first multidimensional outcome related trial in Nepal for HIV infected people. Fourth, the intervention process followed extensive quality control and results on all study effects were large. We followed rigorous analysis methods and reported the effect sizes of all the outcomes. Lastly, randomization process, pretested tools, intervention manual and blinding analysis assessor increased the validity and reliability.

This study has several limitations. First, we delivered the intervention in a single study setting and participants were not blinded, which could lead to contamination. However, we detected highly significant differences among the intervention and control group at 3- and 6-months after baseline. Second, the intervention was delivered by skilled personnel with public health graduate degrees, thus limited availability of skilled personnel would limit its sustainability and accessibility. Next, the intervention was led by less trained service providers need to be assessed. Third, we did not assess and determine if the benefit of the intervention was sustainable; we only assessed the outcome at 3- and 6-months follow up. Fourth, outcomes of subgroup analyses were difficult to validate due to the small sample size. Finally, we did not cover the economic and biomarker aspects.

## Conclusions

Rigorously designed intervention indicates that empowerment intervention can increase QoL of HIV infected people. Further, it could be useful to reduce stigma and increase their social support network. Findings could be utilized at regular service settings for its sustainability and long-term effect. Although the intervention effects on secondary outcomes were detected, we recommend evaluating in future multicenter studies with large sample sizes for monitoring the long term effects.

## Electronic Supplementary Material

Below is the link to the electronic supplementary material.

Supplementary material 1 (DOCX 16 kb)
