# Supplementary material for: Efficacy of a Social Self-Value Empowerment Intervention to Improve Quality of Life of HIV Infected People Receiving Antiretroviral Treatment in Nepal: A Randomized Controlled Trial
Source: AIDS Behav. 2016 Sep 9;21(6):1620–31. doi: 10.1007/s10461-016-1546-z (PMC5422450; doi:10.1007/s10461-016-1546-z)
Supplement: Supplementary file 1 — Supplementary material 1 (DOCX 16 kb) [file 10461_2016_1546_MOESM1_ESM.docx]

**Additional Table 1 Contents of the empowerment intervention**

| **Session** | **Goals** |
| --- | --- |
| **Session 1: Rapport building, living with emotions, mind, sentiments, anger and its management** | - Identify comfortable and uncomfortable situations - Discuss part events and pity for healing to participants’ history - Discuss feelings and experiences within group - Produce knowledge and build up abilities in emotions and sentiments - Identify all the tension and produce idea to cope with tension/stress/righteous anger reveal unnecessary thinking and circumstances and fight with negative thoughts. - Good thinking and things living with HIV, irritations and its types, and learn alternatives to irritations. |
| **Session 2: Meaning of HIV positive, self-esteem and disclosure** | - Discuss the meaning of self-respect/self-confidence/self-esteem - Perform the self-respect actions that discover the aspects of one’s personality. - Strategies, barriers and facilitate to safe disclosure - Develop knowledge in stigma, study the character of stigma in one’s life and discuss how to defeat with it. |
| **Session 3: Healthy body after being HIV positive and control over the future** | - Produce knowledge in sexual, sentimental, emotional and physical areas. Optimism and control over the future. - Explore what it mean to be HIV positive or negative to be a men or women - Discuss about treatment, care after positive, sexuality and barriers. - Discuss transmission and prevention for being a healthy sexual person |
| **Session 4: Relationship, autonomy and community activism** | - Produce knowledge and build up abilities in the social/community/family areas, autonomy and community activism. - Find out the distinction among harmful and healthy relationships. - Discuss the way to identify and cope with harmful relationship. - Develop strategies to establish effective communications with the wishes, sentiments and desires. - Planning the social and family role maintain. |
| **Session 5: Drug, self care and relationships** | - Produce knowledge and build up abilities in the sexual, social areas - Perform excellent communication and listening abilities - Aware of opportunistic infections, other diseases and partners sexual behaviors - Discuss the impacts of drug, alcohol, and smoking to one’s life. - Eating habits, diets, and exercise effects on one’s life |
| **Session 6: Relationship, stress, stigma, discrimination, power-powerlessness, legal empowerment** | - Discuss workplace difficulties, discrimination, power-powerlessness, legal protection, health and human right. - Formulate the objectives for the future - Discuss to join the social and community activities, strategies to raise voice - Find out the situation that you got tension and practice methods to reduce stress and worries. - Conclude and sum up the intervention program |
